# Some Explicit Solutions to the Three-Dimensional Nonlinear Water Wave Problem

**DOI:** 10.1007/s00021-021-00564-4

**Published:** 2021-03-01

**Authors:** Calin I. Martin

**Affiliations:** Faculty of Mathematics, University of Vienna, Oskar-Morgenstern-Platz 1, 1090 Vienna, Austria

**Keywords:** Three-dimensional water-wave problem, Explicit solutions, 35Q31, 35Q35, 76B15, 76U05

## Abstract

We present some explicit solutions (given in Eulerian coordinates) to the three-dimensional nonlinear water wave problem. The velocity field of some of the solutions exhibits a non-constant vorticity vector. An added bonus of the solutions we find is the possibility of incorporating a variable (in time and space) surface pressure which has a radial structure. A special type of radial structure of the surface pressure (of exponential type) is one of the features displayed by hurricanes, cf. Overland (Earle, Malahoff (eds) Overland in ocean wave climate, Plenum Pub. Corp., New York, 1979).

## Introduction

The ubiquitous manifestation of water wave propagation has been given mathematical attention as early as the eighteenth century through the works of Bernoulli, Euler, Lagrange and d’Alembert. The latter works, followed in the nineteenth century by the major achievements of Navier and Stokes, have immensely boosted the development of the mathematical sciences. However, the field of fluid dynamics is far from having left no stone unturned. Notably, one drawback is the pronounced shortage of explicit solutions to the governing equations of water flows with a non-flat free surface.

In the absence of explicit solutions, most exact solutions pertaining to surface water wave propagation (and the flow beneath) were obtained by perturbation approaches employed to replace the nonlinear governing equations by (linear or nonlinear) approximate models which constituted the basis for an assortment of theoretical studies.

A remarkable explicit solution pertaining to pure gravity water waves (over two-dimensional flows of infinite depth) was presented (in Lagrangian coordinates) by Gerstner in 1802, cf. [16]. While being rediscovered subsequently by Froude [15] and Rankine [34], Gerstner’s solution remained until today the only known non-trivial solution satisfying the nonlinear two-dimensional water wave problem. An explicit solution in Eulerian coordinates to the nonlinear two-dimensional water wave problem is still missing.

However, the first rigorous mathematical analysis of the Gerstner wave was performed by Constantin [2], who, by an interplay of topological and analytical arguments, showed that the evolution of the fluid domain under the passage of Gerstner’s wave is consistent with the governing equations.

By means of an essential and relevant extension of the Gerstner wave solution, Constantin has constructed explicit solutions in Lagrangian coordinates [4–7] that portray three-dimensional geophysical flows with a preferred propagation direction. The skeleton of the Gerstner wave was used to further the study of geophysical fluid dynamics [1, 11, 18, 19, 22–24, 28, 30–32].

While the Lagrangian perspective conveys important insights into the flow evolution by tracking down the path of particular particles, it is also desirable to know the velocity field, the pressure function as well as the shape of the free surface at any given time instant and physical location, prospect known as the Eulerian picture. Meeting the latter demand are the studies of Constantin and Johnson [10, 12] presenting exact solutions in Eulerian coordinates which describe three-dimensional ocean flows. Exact and/or explicit solutions in fluid mechanics are extremly rare and, while, at times, lacking practicality, they might confirm the correctness of the governing equations. On the other hand, they supply the foundations of more direct and relevant analyses by means of asymptotic or perturbative methods.

In line with the previous ideas we present in this paper some explicit solutions (in Eulerian coordinates) to the nonlinear three-dimensional water wave problem. We would like to remark that, while the Gerstner wave solution is explicit only concerning the velocity field and the pressure, our solutions are explicit also in terms of the free surface. Furthermore, a spin-off of these solutions is that they also accommodate a variable pressure on the free surface of the constructed flows. The surface pressure distribution of the type we consider here appears to be of relevance in modeling the event of storms in the ocean, cf. [33]. On a related note, we remark that the adjustment of surface pressure conditions seems to be a key-mechanism that leads to the appearance of critical layers, cf. [27].

After we introduce the physical problem we start in Sect. 3 with deriving the explicit solutions to the full nonlinear water wave problem. To our best knowledge this type of solutions were not presented elsewhere before. It is interesting to note that, once the velocity field of the solutions we obtain are written in spherical coordinates, it recovers the velocity field of recent solutions [8, 9, 20, 21] concerning water flows exhiting a preferred propagation direction–the azimuthal one–with no variation in this direction. The benefit of the solutions presented here is that the free surface is explicitly given.

## The Equations of Motion

We describe here the nonlinear water wave problem with free boundary conditions. Working in a Cartesian coordinate system of coordinates *x*, *y*, *z*, we assume the water flow to be bounded below by the bed $z=-d$ and above by the free surface $z=\eta (x,y,t)$, where $\eta$ is a function that is determined as part of the solution and *t* denotes the time variable.

Assuming the fluid to be homogeneous, of constant density $\rho$–a reasonable assumption, cf. [3, 14]–we have the equation of mass conservation in the form

2.1 $$\begin{aligned} u_x+v_y+w_z=0, \end{aligned}$$

where (*u*, *v*, *w*) represents the velocity field. Under the assumption that water is inviscid, the equations of motion are Euler’s equations

2.2 $$\begin{aligned} u_{t}+u u_{x}+v u_{y}+w u_{z}&= -\frac{1}{\rho }P_x,\nonumber \\ v_{t}+u v_{x}+v v_{y}+w v_{z}&= -\frac{1}{\rho }P_y,\nonumber \\ w_{t}+u w_{x}+v w_{y}+ w w_{z}&= -\frac{1}{\rho }P_z-g, \end{aligned}$$

where *P* is the pressure function and *g* denotes the gravitational constant.

While both (2.1), (2.2) are required to take place within the bulk of the fluid, the specification of the water wave problem is completed by the boundary conditions pertaining to the free surface $z=\eta (x,y,t)$ and to the bed $z=-d$. These are the kinematic boundary conditions

2.3 $$\begin{aligned} w=\eta _t +u\eta _x+v\eta _y \quad {{\mathrm{on}}}\quad z=\eta (x,y,t) \end{aligned}$$

and

2.4 $$\begin{aligned} w=0\quad {{\mathrm{on}}}\quad z=-d, \end{aligned}$$

together with the dynamic boundary condition

2.5 $$\begin{aligned} P=P_{atm}\quad {{\mathrm{on}}}\quad z=\eta (x,y,t), \end{aligned}$$

where $P_{atm}$ denotes the constant atmospheric pressure. Condition (2.5) decouples the motion of the water from the motion of the air above it, cf. Constantin [3].

We are now ready to introduce a family of time independent solutions.

## Explicit Solutions

While in Sect. 3.1 we present a family of time-independent solutions to the water wave problem (2.1)–(2.5), Sect. 3.2 displays time-dependent solutions of traveling type. The discussion is extended in both Sects. 3.1 and 3.2 to the case where the dynamic boundary condition (2.5) is replaced to a condition that requires that the pressure on the free surface equals some more general prescribed function, of radial type (in the horizontal variables *x*, *y*).

### A Family of Time Independent Solutions

We start by making the Ansatz

3.1 $$\begin{aligned} u(x,y,z)=-yf(x^2+y^2),\quad v(x,y,z)=xf(x^2+y^2)\,\, {{\mathrm{and}}}\,\,w(x,y,z)=0, \end{aligned}$$

where $f:{\mathbb {R}}\rightarrow {\mathbb {R}}$ is such that the functions $(x,y)\rightarrow -yf(x^2+y^2)$ and $(x,y)\rightarrow x f(x^2+y^2)$ are differentiable. A computation shows that

3.2 $$\begin{aligned} \begin{aligned} u_t+uu_x+vu_y&=-xf^2(x^2+y^2)\\ v_t+uv_x+vv_y&=-yf^2(x^2+y^2). \end{aligned} \end{aligned}$$

This means that, in order to find the pressure *P* we have to solve the system

3.3 $$\begin{aligned} \begin{aligned} \frac{1}{\rho }P_x(x,y,z,t)&=xf^2(x^2+y^2)\\ \frac{1}{\rho }P_y(x,y,z,t)&=yf^2(x^2+y^2)\\ \frac{1}{\rho }P_z(x,y,z,t)&=-g. \end{aligned} \end{aligned}$$

Solving for *P* in the above system we obtain

3.4 $$\begin{aligned} P(x,y,z,t)=\rho \left( \frac{1}{2}\int _0^{x^2+y^2}f^2(s)ds-gz+c(t)\right) , \end{aligned}$$

where $t\rightarrow c(t)$ is some function depending only on *t*. Using the dynamic surface condition (2.5) we determine the free surface $\eta$ by

3.5 $$\begin{aligned} \eta (x,y,t)=\frac{1}{g}\left( \frac{1}{2}\int _0^{x^2+y^2}f^2(s)ds +c(t)-\frac{P_{atm}}{\rho }\right) . \end{aligned}$$

From formula (3.5) we obtain, by glancing also at the expressions of *u* and *v*, that

3.6 $$\begin{aligned} \eta _t(x,y,t)+u\eta _x(x,y,t)+v\eta _y(x,y,t)=\frac{c^{\prime }(t)}{g}\,\,\mathrm{for}\,\,\mathrm{all}\,\,x,y,t. \end{aligned}$$

Choosing now $c^{\prime }(t)=0$ for all *t*, we see from (3.6) that the surface kinematic condition (2.3) is satisfied.

We can summarize the previous calculations in the following Theorem.

#### Theorem 3.1

The tuple $(u,v,w,P,\eta )$ given in (3.1), (3.4) and (3.5), respectively, represents an explicit solution to the water wave problem (2.1)–(2.5).

#### Remark 3.2

While apparently absent in (3.1), the vertical structure of the flow we derived becomes conspicuous when the velocity field is written in spherical coordinates. Indeed, let *r* denote the distance from a point in the flow to the center of the Earth, $\theta$ denotes the polar angle, i.e. the angle between the *z* axis and the and the radial vector connecting the origin to the point in question and $\phi$ represents the azimuthal angle. Then a point (*x*, *y*, *z*) is written in spherical coordinates as

3.7 $$\begin{aligned} \begin{aligned} x&= r\sin \theta \cos \phi \\ y&= r\sin \theta \sin \phi \\ z&= r\cos \theta . \end{aligned} \end{aligned}$$

Moreover, the basis unit vectors ${\mathbf {e}}_r,{\mathbf {e}}_{\theta }, {\mathbf {e}}_{\phi }$ are related to the cartesian unit vectors $e_1,e_2, e_3$ as

3.8 $$\begin{aligned} \begin{aligned} e_1&=(\sin \theta \cos \phi ){\mathbf {e}}_r +(\cos \theta \cos \phi ){\mathbf {e}}_{\theta }-(\sin \phi ){\mathbf {e}}_{\phi }\\ e_2&=(\sin \theta \sin \phi ){\mathbf {e}}_r +(\cos \theta \sin \phi ){\mathbf {e}}_{\theta }+(\cos \phi ){\mathbf {e}}_{\phi }\\ e_3&=(\cos \theta ){\mathbf {e}}_r-(\sin \theta ){\mathbf {e}}_{\theta }. \end{aligned} \end{aligned}$$

Therefore

3.9 $$\begin{aligned} \begin{aligned}&(-yf(x^2+y^2),xf(x^2+y^2),0)\\&\quad =-(r\sin \theta \sin \phi )f(r^2\sin ^2\theta )[(\sin \theta \cos \phi ){\mathbf {e}}_r +(\cos \theta \cos \phi ){\mathbf {e}}_{\theta }-(\sin \phi ){\mathbf {e}}_{\phi }]\\&\qquad +(r\sin \theta \cos \phi )f(r^2\sin ^2\theta ) [(\sin \theta \sin \phi ){\mathbf {e}}_r +(\cos \theta \sin \phi ){\mathbf {e}}_{\theta }+(\cos \phi ){\mathbf {e}}_{\phi }]\\&\quad =(r\sin \theta )f(r^2\sin ^2\theta ){\mathbf {e}}_{\phi }, \end{aligned} \end{aligned}$$

that is, in spherical coordinates, the velocity field describes a flow that moves only in the azimuthal direction without any variation in this direction. It was shown by Constantin and Johnson [8, 9] (see also [20, 21, 29] that the only ocean flows that propagate in the azimuthal direction with no variation in this direction are those that exhibit a velocity profile of the type $F(r\sin \theta ){\mathbf {e}}_{\phi }$, where $(r,\theta )\rightarrow F(r\sin \theta )$ is some arbitrary differentiable function.

The considerations in this remark hint to the possibility that some of the features displayed by the ocean flows (like the pronounced vertical structure) are better seized through the involvement of spherical coordinates. However, while the free surface of the flows presented in this paper in cartesian coordinates are explicitly given (cf. (3.14),(3.22), (3.25)), the surface of the flows in [8, 20, 21] is provided only in terms of an existence type result by means of an implicit function theorem. For a selective list of works presenting exact solutions to the water wave problem with Coriolis and centripetal terms we refer the reader to [5, 6, 8, 9, 18, 20–22, 30].

#### Remark 3.3

The vorticity of the flow (3.1) is

3.10 $$\begin{aligned} (0,0,v_x-u_y)=\left( 0,0,2f(x^2+y^2)+2(x^2+y^2)f^{\prime }(x^2+y^2)\right) , \end{aligned}$$

which is non-constant for an appreciable number of functions *f* with the property required in (3.1). Furthermore, the rotationality of the flow (3.1), (3.4), (3.5) appears to constitute a marked difference from three-dimensional water flows obtained by means of existence type results [13, 17, 25, 26, 35].

#### Remark 3.4

We allow now a variable (in space and time) pressure at the surface. That is, we maintain the velocity field (3.1), but, instead of (2.5), we require

3.11 $$\begin{aligned} P(x,y,\eta (x,y,t),t)=h(t)+F(x^2+y^2),\,\,{{\mathrm{for}}}\,\,{{\mathrm{all}}}\,\,x,y,t, \end{aligned}$$

where $t\rightarrow h(t)$ is differentiable and *F* is an arbitrary function such that $(x,y)\rightarrow F(x^2+y^2)$ is differentiable. With the imposed pressure (3.11) at the surface we have that the expression of the free surface is now

3.12 $$\begin{aligned} \eta (x,y,t)=\frac{1}{g}\left( \frac{1}{2}\int _0^{x^2+y^2}f^2(s)ds +c(t)-\frac{h(t)+F(x^2+y^2)}{\rho }\right) \,\,{{\mathrm{for}}}\,\,{{\mathrm{all}}}\,\,x,y,t. \end{aligned}$$

To check that the kinematic boundary condition (2.3) is still in place, we notice that

3.13 $$\begin{aligned} \eta _t(x,y,t)+u\eta _x(x,y,t)+v\eta _y(x,y,t)=\frac{1}{g} \left( c^{\prime }(t)-\frac{h^{\prime }(t)}{\rho }\right) \,\,{{\mathrm{for}}}\,\,{{\mathrm{all}}}\,\,x,y,t, \end{aligned}$$

and all we have to do now is to choose $c(t)=\frac{h(t)}{\rho }+a$, where *a* is some constant. The formula for the free surface becomes

3.14 $$\begin{aligned} \eta (x,y,t)=\frac{1}{g}\left( \frac{1}{2}\int _0^{x^2+y^2}f^2(s)ds -\frac{F(x^2+y^2)}{\rho }+a\right) \,\,{{\mathrm{for}}}\,\,{{\mathrm{all}}}\,\,x,y,t. \end{aligned}$$

### A Time Dependent Solution

Let

3.15 $$\begin{aligned} u(x,y,z,t)=t-y,\quad v(x,y,z,t)=-t+x,\quad w\equiv 0. \end{aligned}$$

Clearly, the equation of mass conservation (2.1) is satisfied. Furthermore, the Euler equations are equivalent to

3.16 $$\begin{aligned} \begin{aligned} \frac{1}{\rho }P_x&=x-1-t\\ \frac{1}{\rho }P_y&=y+1-t\\ \frac{1}{\rho }P_z&=-g. \end{aligned} \end{aligned}$$

Hence, the pressure is given by the general formula

3.17 $$\begin{aligned} P(x,y,z,t)=\rho \left( \frac{(x-1)^2+(y+1)^2}{2}-t(x+y)-gz+c(t)\right) , \end{aligned}$$

where $t\rightarrow c(t)$ is some function that depends only on *t*, which will be chosen in a convenient way later on. From the dynamic boundary condition we infer that

3.18 $$\begin{aligned} \eta (x,y,t)=\frac{1}{g}\left( \frac{(x-1)^2+(y+1)^2}{2}-t(x+y)+c(t )-\frac{P_{atm}}{\rho }\right) . \end{aligned}$$

We see now from (3.18) that

3.19 $$\begin{aligned} g(u\eta _x+v\eta _y)=(t-y)(x-1-t)+(-t+x)(y+1-t)=-2t+x+y \end{aligned}$$

and also

3.20 $$\begin{aligned} g\eta _t(x,y,t)=-(x+y)+c^{\prime }(t), \end{aligned}$$

from which we infer that

3.21 $$\begin{aligned} \eta _t+u\eta _x+v\eta _y=\frac{1}{g}\left( c^{\prime }(t)-2t\right) =0 \end{aligned}$$

if $c(t)=t^2$. Since $w\equiv 0$ we have that the kinematic surface condition (2.3) is verified. We thus have the following:

#### Theorem 3.5

The tuple $(u,v,w,P,\eta )$ given in (3.15), (3.17) and (3.18), respectively, represents a time-dependent solution to the water wave problem (2.1)–(2.5).

#### Remark 3.6

Note that with $c(t)=t^2$ the formula for $\eta$ becomes

3.22 $$\begin{aligned} \eta (x,y,t)&=\frac{1}{g}\left( \frac{(x-t)^2+(y-t)^2}{2}-x+y+1-\frac{P_{atm}}{\rho }\right) \nonumber \\&=\frac{1}{g}\left( \frac{(x-t)^2+(y-t)^2}{2}-(x-t)+y-t+1-\frac{P_{atm}}{\rho }\right) , \end{aligned}$$

that is, the free surface has a steady like behaviour in both horizontal directions.

#### Remark 3.7

As in the previous example, we set out to discuss the case of a variable pressure on the free surface $z=\eta (x,y,t)$. We keep the velocity field as in (3.15), but allow a variable pressure on the free surface, that is we ask

3.23 $$\begin{aligned} P(x,y,\eta (x,y,t),t)=k(t)+G(x^2+y^2),\,\,\quad {{\mathrm{for}}}\,\,{{\mathrm{all}}}\,\,x,y,t, \end{aligned}$$

(where $t\rightarrow k(t)$ and $(x,y)\rightarrow G(x^2+y^2)$ are some differentiable, but otherwise, arbitrary functions) we see that the free surface is given as

$$\begin{aligned} \eta (x,y,t)=\frac{1}{g}\left( \frac{(x-1)^2+(y+1)^2}{2}-t(x+y)+c(t) -\frac{k(t)+G(x^2+y^2)}{\rho }\right) . \end{aligned}$$

It turns out that

3.24 $$\begin{aligned} \eta _t+u\eta _x+v\eta _y=\frac{1}{g}\left( c^{\prime }(t) -\frac{k^{\prime }(t)}{\rho }-2t\right) . \end{aligned}$$

Choosing now $c(t):=\frac{k(t)}{\rho }+t^2+b$, for some constant $b\in {\mathbb {R}}$, we obtain that

$$\begin{aligned} \eta _t+u\eta _x+v\eta _y=0=w, \end{aligned}$$

that is the kinematic surface condition is satisfied. Moreover, the free surface is given in this case as

3.25 $$\begin{aligned} \begin{aligned} \eta (x,y,t) =\frac{1}{g}\left( \frac{(x-t)^2+(y-t)^2}{2}-(x-t)+y-t-\frac{G(x^2+y^2)}{\rho }+b\right) . \end{aligned} \end{aligned}$$
